# Supplementary material for: Vascular network-inspired fluidic system (VasFluidics) with spatially functionalizable membranous walls
Source: Nat Commun. 2024 Feb 16;15:1437. doi: 10.1038/s41467-024-45781-3 (PMC10873510; doi:10.1038/s41467-024-45781-3)
Supplement: Supplementary file 1 — Supplementary Information [file 41467_2024_45781_MOESM1_ESM.pdf]

Supplementary Information

Vascular network-inspired fluidic system (VasFluidics) with spatially functionalizable membranous walls

Yafeng Yu[1], Yi Pan[1,3], Yanting Shen[1], Jingxuan Tian[1,2], Ruotong Zhang[1], Wei Guo[1,2], Chang Li[1], and Ho Cheung Shum[1,2]*

[1] Department of Mechanical Engineering, The University of Hong Kong, Pokfulam Road, Hong Kong (SAR), China. [2] Advanced Biomedical Instrumentation Centre, Hong Kong Science Park, Shatin, New Territories, Hong Kong (SAR), China. [3] Present address: Institute of Biomedical Engineering, College of Medicine, Southwest Jiaotong University, 610031 Chengdu, China. *Email: ashum@hku.hk

Supplementary Note 1: Rheological properties of chitosan printing ink and APAM printing matrix

The chitosan printing inks are shear-thinning with zero-shear viscosities higher than 0.5 Pa·s, as shown in Supplementary Fig. 2(a). The ink with shear-thinning property can be controllably extruded from the nozzle[1,2], since the viscosity decreases under increased shear rate. The ink with appropriate viscosities can be extruded into threads instead of breaking up into droplets during ink deposition[1,3]. In our experiment, coacervates self-assemble on the ink-matrix interface, locking chitosan polymers inside before they spread around.

The APAM matrix is a shear-thinning and viscoelastic liquid, as demonstrated in Supplementary Fig. 2(b, c). The shear-thinning matrix with a low-yielding stress (≤ 10 Pa) allows the print nozzle to move freely during printing[1]. In addition, the elastic modulus G' of the matrix is higher than the viscous modulus G" at low shear stress; hence, the matrix is more solid-like. The solid-like matrix can facilitate the stabilization of printed structures after ink deposition[1,4].

Supplementary Note 2: Controllable size of printed channels

The size of printed channels is determined by the channel length and the area of cross-section perpendicular to the length. The channel length is decided by the printing distance *D* (unit: mm), as shown in Supplementary Fig. 9(a). The area of the cross-section perpendicular to the length is defined as a (mm^2), which can be adjusted by changing the printing speed *P* (mm s^{-1}) and the flow rate of printing ink (*F*, μL s^{-1}) (Supplementary Fig. 9(a)). Specifically, printing speed *P* refers to the moving speed of the print nozzle, determined by the print distance (*D*, unit: mm) of the nozzle per unit of printing time (*T*, unit: s):

$$P = \frac{D}{T} \tag{1}$$

Ink flow rate *F* determines the ink volume (unit: mm^3) being extruded from the print nozzle per unit of printing time *T*, which relates to the volume of the printed channel:

$$FT = Da \tag{2}$$

Equation 1 and Equation 2 figure out the theoretical relationship between a, *F*, and *P*:

$$a = \frac{F}{P} \tag{3}$$

By measuring channels with different cross-sectional areas (Supplementary Fig. 9(c)), we find the measured *a* matches well with Equation 3 (Supplementary Fig. 9(b)). Therefore, *a* of printed channels is predictable with known *F* and *P* (Equation 3).

Morphological parameters of the cross-sections can be further estimated with known values of a. We approximated morphologies of channels' cross-sections as parts of a circle, as shown in Supplementary Fig. 10(a). The height *h*, the width *w*, the length of the membrane part *m*, and the length that channel attached to the substrate *s* were measured by analyzing confocal laser scanning images of channels' cross-sections in Supplementary Fig. 9(c). Since parameters of lengths (*m*, *w*, *s*, *h*) should have approximate square relation with the area (*a*), we approximated the relation between *h* (or *w*, *m*, *s*) and *a* using the following Equation 4, which fits the measured data well (Supplementary Fig. 10(b)):

$$h(or\ w, m, s) = k\sqrt{a} \tag{4}$$

where *k* is a constant. In this equation, *w* equals *s* with a constant value of 520-620 μm when *a* ≤ 0.1 mm^2. The constant value relates to the size of the print nozzle tip (inner diameter =

389.9±4.5 μm, external diameter = 851.3±27.3 μm), and the average value of its inner and outer diameters is 620.6 μm. Hence, channels with smaller *w* are unavailable with the such large-sized print nozzle. Since the value of *w* can be directly measured under an optical microscope, *a*, *h*, and *m* can also be estimated using measured *w* when *w* is larger than 620 μm, as indicated in Equation 4. Moreover, we found volumes of channels expand with the prolonged assembly time of membranes (T_{ma}), which can lead to estimation errors in Equation 3 and Equation 4. The change ratios of *a*, *h*, *m*, *w*, *s* are present in Supplementary Fig. 11. The volumetric expansion may result from the unbalanced osmotic pressure between the printing ink and matrix.

By utilizing the pipet tip-based nozzle tip, channels we obtained have a widths (*w*) of around 500 μm-2 cm. Larger channels are not obtained due to the limitations of our setups. For instance, the printing speed cannot be decreased further with our current 3D printer, and our current pump cannot provide a higher pressure to extrude the viscous printing ink at a higher ink flow rate. Smaller channels can also be printed by using printing nozzle tip with a smaller diameter, for instance, as presented in Supplementary Fig. 12, thin channels with *w* around 200 μm are printed with a fine stainless steel needle (inner diameter = 129.5±1.5 μm, external diameter = 245.9±1.7 μm). Hence, although not realized by our setups yet, the fabrication of even larger or smaller sized channels is possible with modified 3D printing setups.

Supplementary Note 3: A dimensionless number to guide possible flow rates in different sized channels

To guide the relationship between the maximum allowable flow rates and the channel size, we derive a governing dimensionless number χ based on the channel size. We approximate the channel cross section as a circle with a diameter of d. We model flow in the channel as a pressure driven pipe flow, where the inlet flow rate results in a flow pressure on the channel wall. The maximum flow rate is reached when the flow-induced shear pressure on the channel wall exceeds the bonding strength between the channel and the substrate.

Under flow rate Q, the shear pressure on the channel wall is

$$\tau_{wall} = \frac{128\mu Q}{4\pi d^3}$$

where μ is fluid viscosity.

The flow induced axial pressure P leads to the expansion of the soft channel, with the diameter expanding from d_0 to $d_0 + \Delta d$. For elastic materials, Δd correlates with d_0 via the Young's modulus E

$$\Delta d = \frac{P}{E} d_0$$

At the maximum flow rate above which the channel will detach from the substrate, the shear stress on the channel wall reaches an equilibrium with the channel-substrate adhesion strength, which gives

$$\frac{128\mu Q_{max}}{4\pi (d_0 + \Delta d_{max})^3} = E_{bond}$$

Assume $E_{bond} = \beta E_0$ and $\Delta d_{max} = \alpha d_0$, where α and β are prefactors, and E_0 is the unit adhesion strength between chitosan and Petri dish.

Herein we have

$$\frac{128\mu Q_{max}}{4\pi (d_0 + \alpha d_0)^3} = \beta E_0$$

Or

$$\frac{128\mu Q_{max}}{4\pi\beta(1+\alpha)^3 E_0 d_0^3} = 1$$

Note that the prefactors α and β could be constant, or dependent on the channel diameter d. In any case, we introduce a shape factor function for the channel $\lambda(d) = \beta(1+\alpha)^3$. And we derive a dimensionless number that measures the ratio between the flow-induced shear stress on the channel wall and the adhesion strength of the channel on the substrate

$$\chi = \frac{128\mu Q}{4\pi\lambda E_0 d_0^3}$$

At the maximum flow rate of $Q{\sim}Q_{max}$ $(Q/Q_{max}{\sim}1)$, we have

$$\chi = \frac{128\mu Q}{4\pi\lambda E_0 d_0^3}{\sim}1$$

It is difficult to accurately estimate $\lambda(d)\ and\ E_0$, as the channel-substrate interaction is complex and their contacting area could be composed of multiplayer of polymers. Nevertheless, we next consider a model case to show the dimensionless parameter χ is effective.

Model case: Considering a weak binding between the channel and the substrate (or between chitosan and Petri dish) with $E_0 =$ 10 mPa, we choose a shape factor function of

$$\lambda(d) = A\left(\frac{d}{d^*}\right)^{\gamma} + B$$

where d^* is a characteristic length scale.

The best fitting of the experimental data in Supplementary Fig. 15c results in that *A* = 199.8, *B* = -143.2, *d** = 0.56 (mm) and γ = 0.5. The fitted curve is plotted in Supplementary Fig.16. In this way, we can calculate χ under different flow conditions, as listed in Supplementary Fig.15c. Apparently, we have $O(0.1) \le \chi \le O(1)$.

To further verify that χ guides the possible flow rates in VasFluidic channels of different sizes, we estimated the maximum flow rate for a channel with a width of 1.2 mm. The

calculated maximum flow rate (924 mL h^{-1}) with χ = 1 is close to the experimental value (1100-1300 mL h^{-1}), below which the channel wall remain intact, with no liquid leakage.

Supplementary Table 1. Dimensions of different substances. As dissolved in water at neutral pH, substances with a radius or hydrodynamic radius (R_h) larger than 3.0 nm cannot pass through the membrane wall within 60 min, while small-sized substances ($R_h \leq 1.9$ nm) can.

**Substances**	**Dimensions**	**Can(√) or cannot(×) pass through channel walls**
Fluorescein sodium salt	$R_h \approx 0.5$ nm [5]	√
R6G (Rhodamine 6G)	$R_h \approx 0.6$ nm [6]	√
FITC-Dex4k (Fluorescein isothiocyanate-labeled dextran, 4,000 g mol^{-1})	$R_h \approx 1.4$ nm [7]	√
FITC-Dex10k (Fluorescein isothiocyanate-labeled dextran, 10,000 g mol^{-1})	$R_h \approx 1.9$ nm [7]	√
FITC-Dex40k (Fluorescein isothiocyanate-labeled dextran, 40,000 g mol^{-1})	$R_h \approx 3.0$ nm [7]	×
FITC-Dex70k (Fluorescein isothiocyanate-labeled dextran, 70,000 g mol^{-1})	$R_h \approx 3.6$ nm [7]	×
GOx (Glucose oxidase)	$R_h \approx 4.5$ nm [8]	×
HRP (Horseradish peroxidase)	$R_h \approx 4$ nm [9]	×
Glucose (Dextrose)	$R_h \approx 0.4$ nm [10]	√
Starch (Maize starch)	Radius ≈ 2.5-10 μm [11]	×

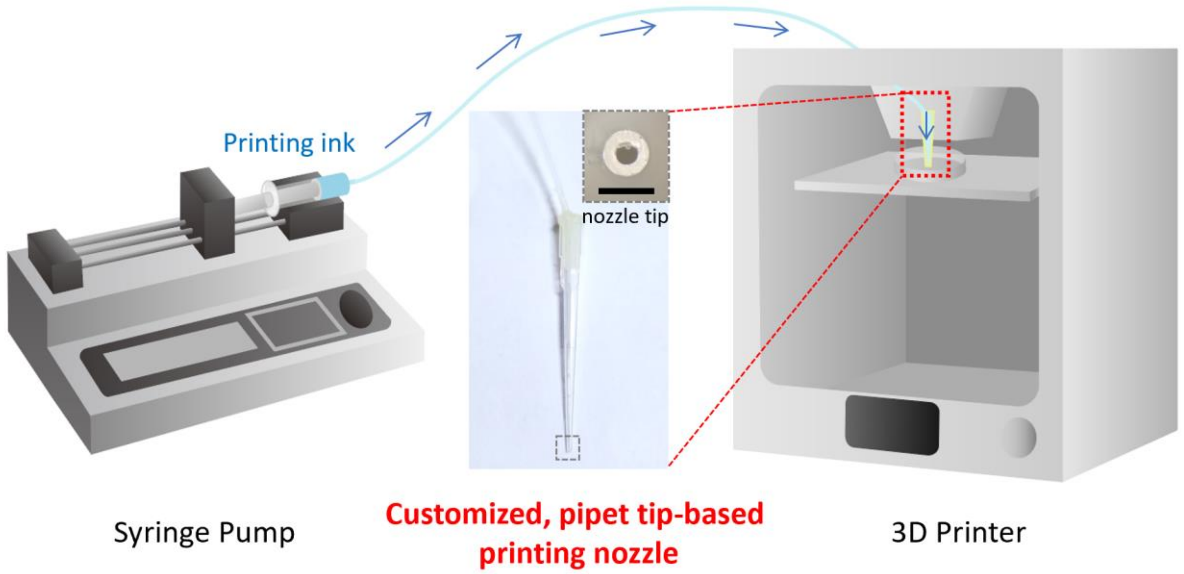

Supplementary Figure 1. Setups for printing channels. A 0.5-10 μL plastic pipette tip is used as a printing nozzle, which is fixed to a 3D printer and connected to a syringe pump. The scale bar is 1 mm.

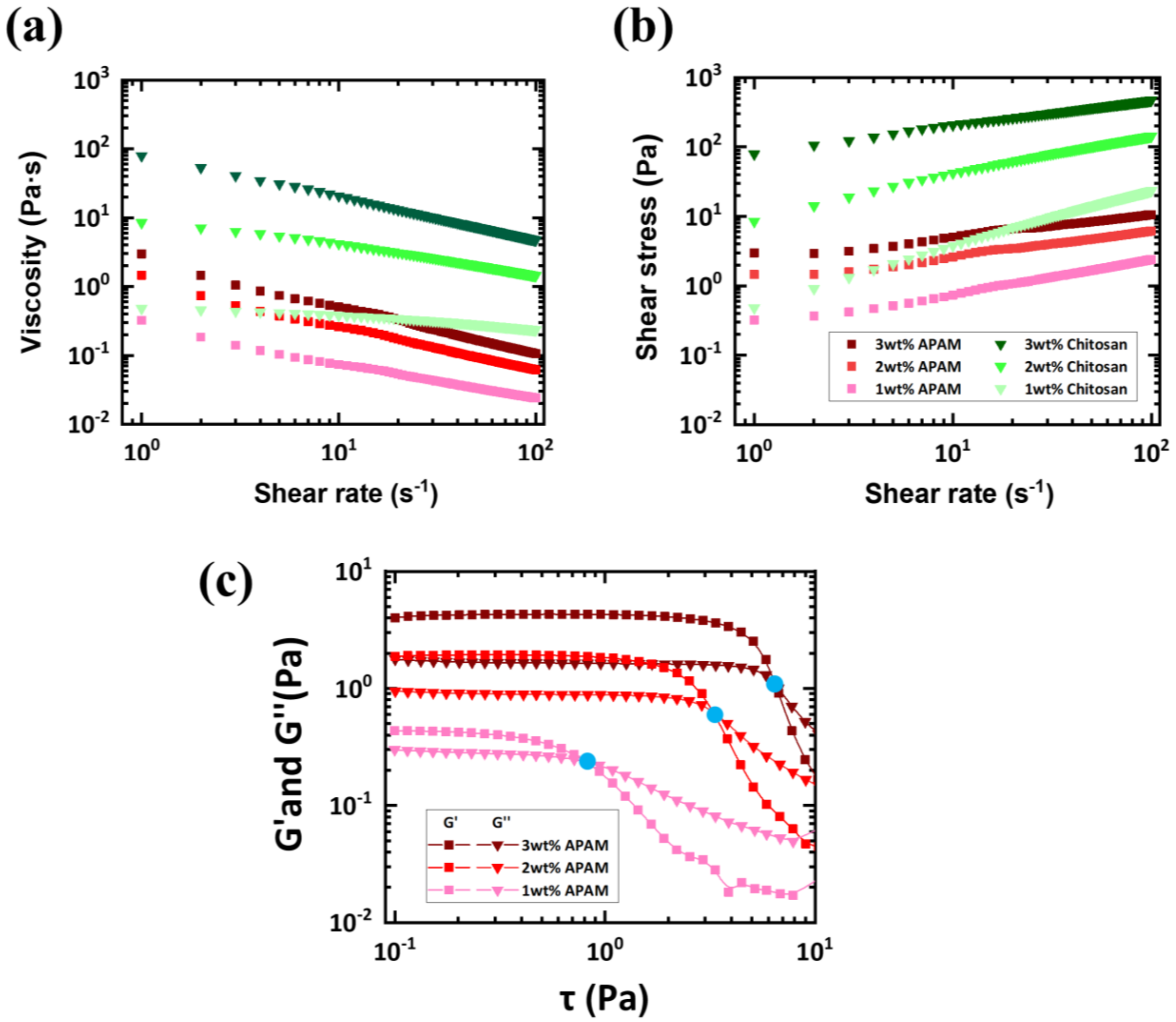

Supplementary Figure 2. The rheological properties of chitosan and APAM aqueous solutions. (a) Viscosity and (b) shear strain against shear rate for 3 wt% APAM solution, 2 wt% APAM solution, 1wt% APAM solution, 3 wt% chitosan solution, 2 wt% chitosan solution and 1 wt% chitosan solution, respectively. The shear rate is set from 10^0 to 10^2, suitable for extrusion scenarios. (c) The elastic, G', and viscous, G'' moduli as a function of the oscillatory shear stress for APAM solutions with different concentrations at a frequency of 1 Hz. The determined yield stress values are circled in blue for each solution. Source data are provided as a Source Data file.

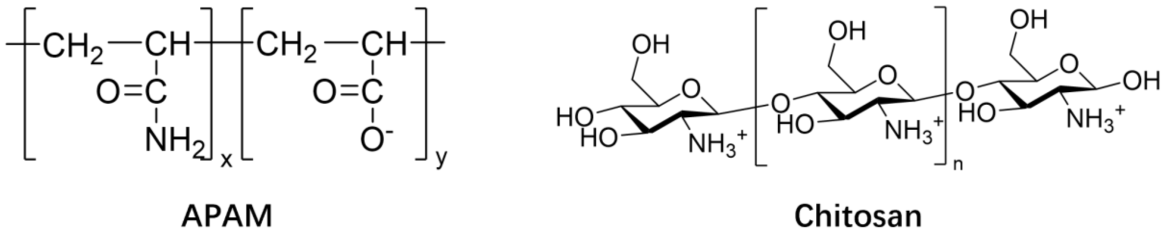

Supplementary Figure 3. Chemical structure of anionic polyacrylamide (APAM) and chitosan. Polymers in APAM aqueous solutions are negatively charged due to the presence of -COO^- groups. The chitosan polymers in acid solution (pH ≤ 6.5) are positively charged due to the protonated amine groups -NH_3^+.

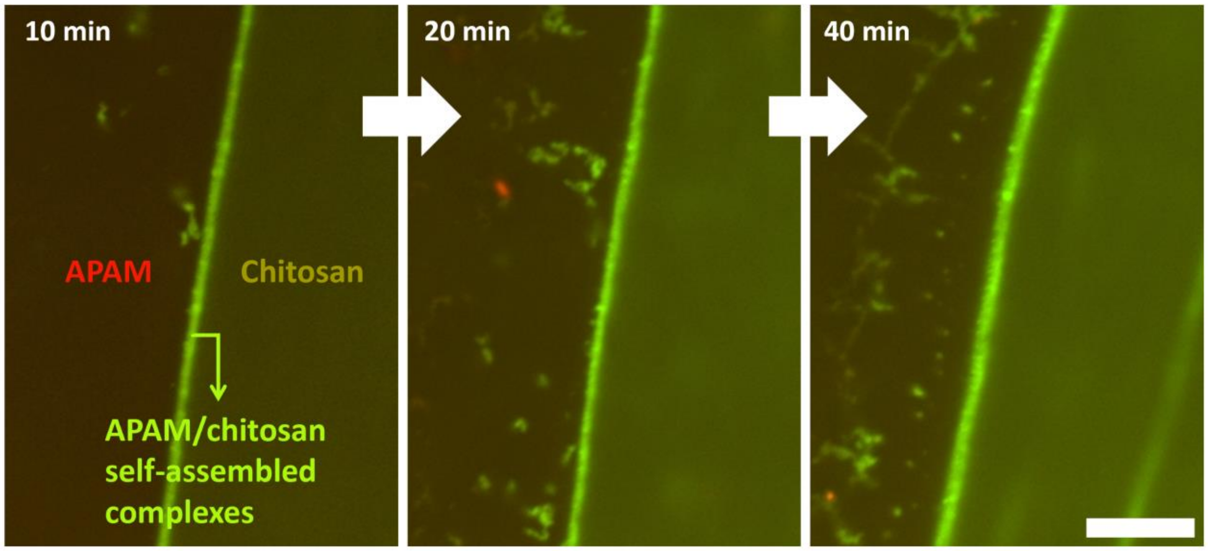

Supplementary Figure 4. Aggregation of polymers on the interface between APAM and chitosan solutions. 1 wt% chitosan solution is pre-mixed with 0.005 wt% fluorescein sodium, and 1 wt% APAM solution is pre-mixed with 0.005 wt% rhodamine 6G for visualization under confocal microscope. The scale bar is 10 μm.

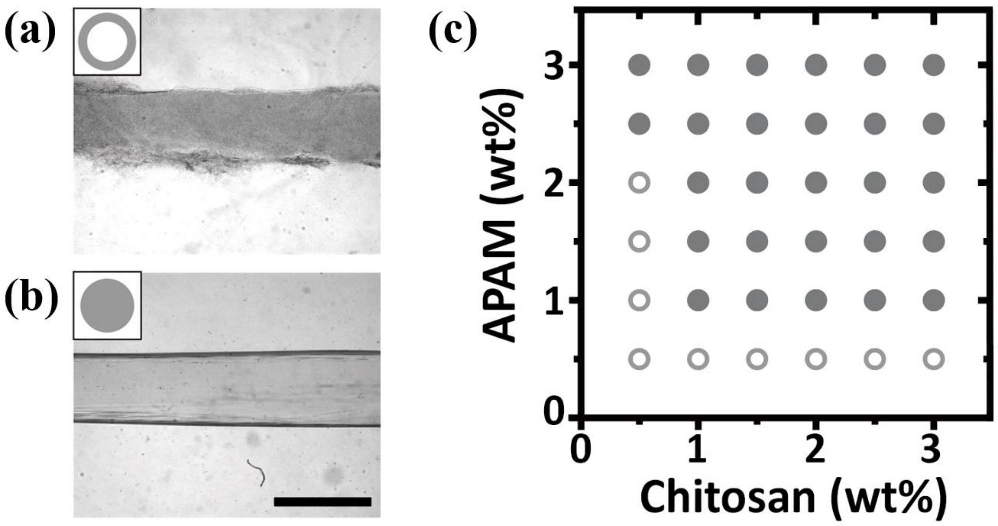

Supplementary Figure 5. Suitable polymer concentrations for printing channel-like chambers. (a) Printed chamber with formless APAM/chitosan wall, marked as hollow circles. (b) Printed channel-like chamber with a membranous APAM/chitosan wall, marked as filled circles. The scale bar is 1 mm. (c) Chitosan and APAM concentration profile for printing chambers in (a) and (b).

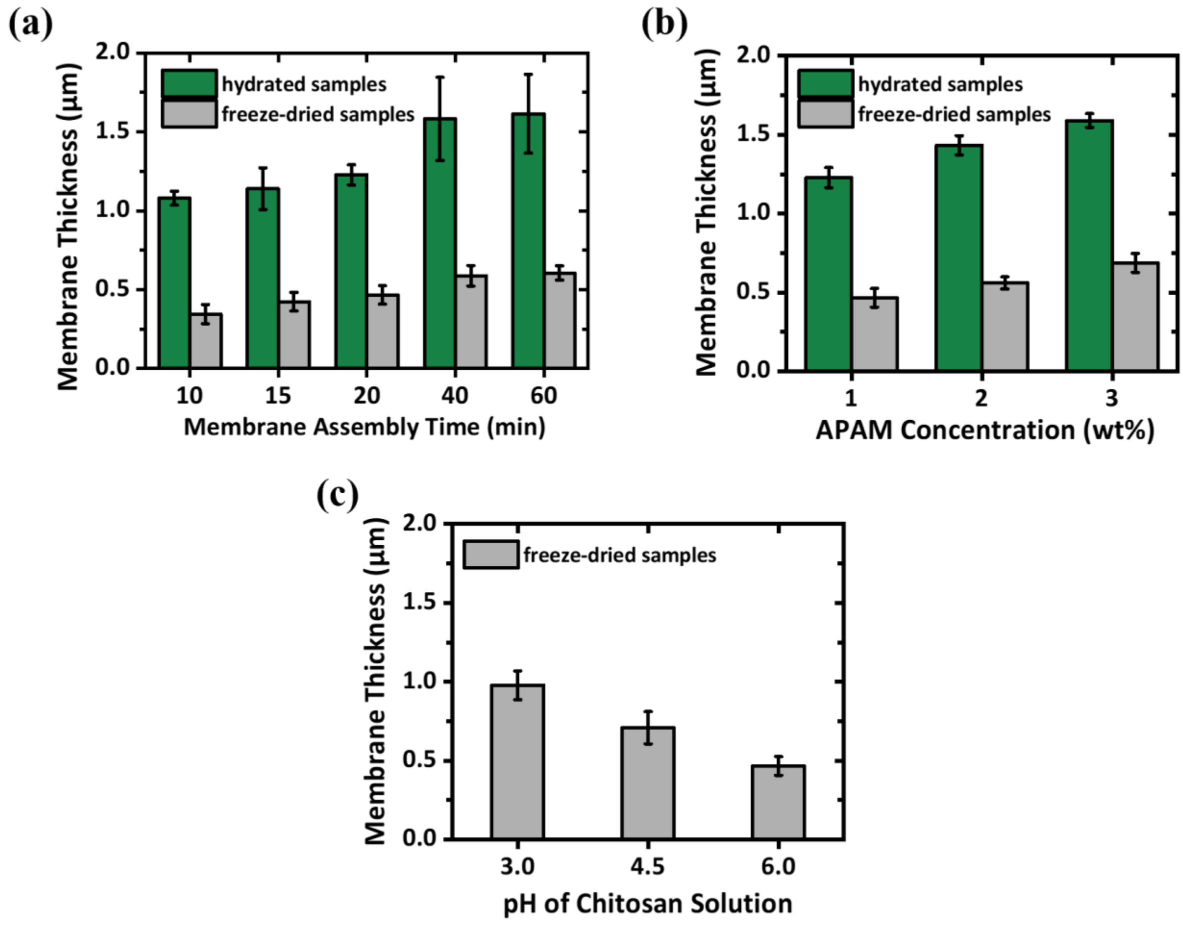

Supplementary Figure 6. Thickness of hydrated and freeze-dried membrane walls. Measurements of hydrated membrane walls may more closely represent their thicknesses as the channels are infused with liquids during operation. The thickness of hydrated membranes is measured with confocal microscope images. The chitosan solution is mixed with 0.005 wt% fluorescein sodium, and the APAM solution is mixed with 0.005 wt% rhodamine 6G for visualizing membrane cross-sections under confocal microscope. The thickness of freeze-dried membranes is measured with scanning electron microscope images. (a) Thicknesses of membranous walls with various membrane assembly time. 1wt% chitosan solution (pH≈6) is used to react with APAM solution (pH≈7) for membrane assembly. (b) Changing thickness of membranes by changing APAM concentrations in APAM printing matrix. 1wt% chitosan solution (pH≈6) is used to react with APAM solution (pH≈7) for membrane assembly, and the duration for membrane assembly is 20 min. (c) Changing thickness of membranes by changing the pH of chitosan printing ink. 1wt% chitosan solutions with different pH are used to react with APAM solution (1wt%, pH≈7) for membrane assembly, and the duration for

membrane assembly is 20 min. Thickness of freeze-dried samples are measured based on the SEM images, and the thickness of hydrated samples are not measured. Hydrated samples are not clearly observed under confocal microscope due to the decreased fluorescence intensity of sodium fluorescein under low pH. Source data are provided as a Source Data file.

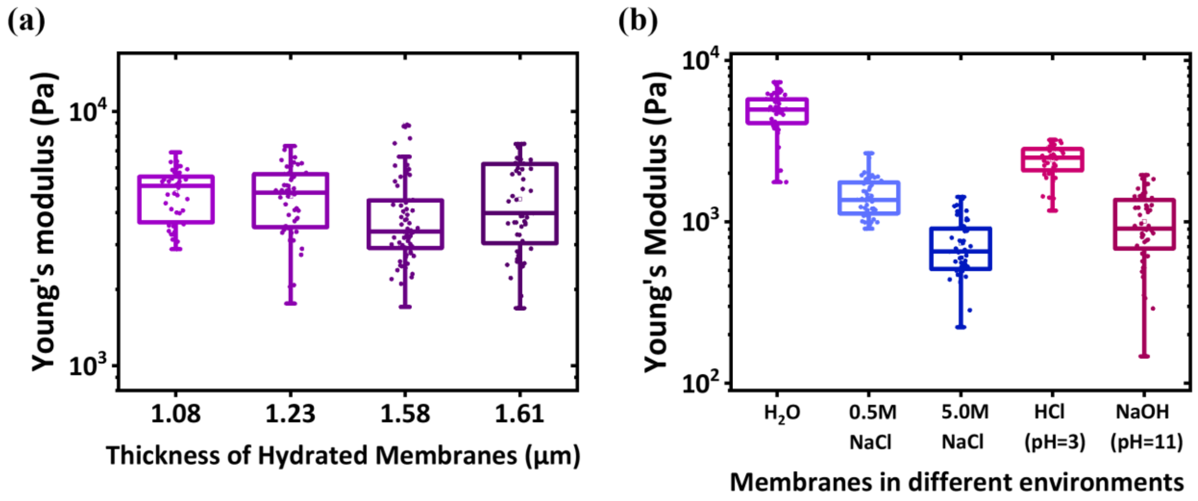

Supplementary Figure 7. (a) Young's modulus of membranous channel walls with different thicknesses. The membranes are immersed under water to stay hydrated during the measurement with atomic force microscope. (b) Young's modulus of membranous channel walls under different environments. Membranes are immersed in different solutions during the measurement with atomic force microscope. Source data are provided as a Source Data file.

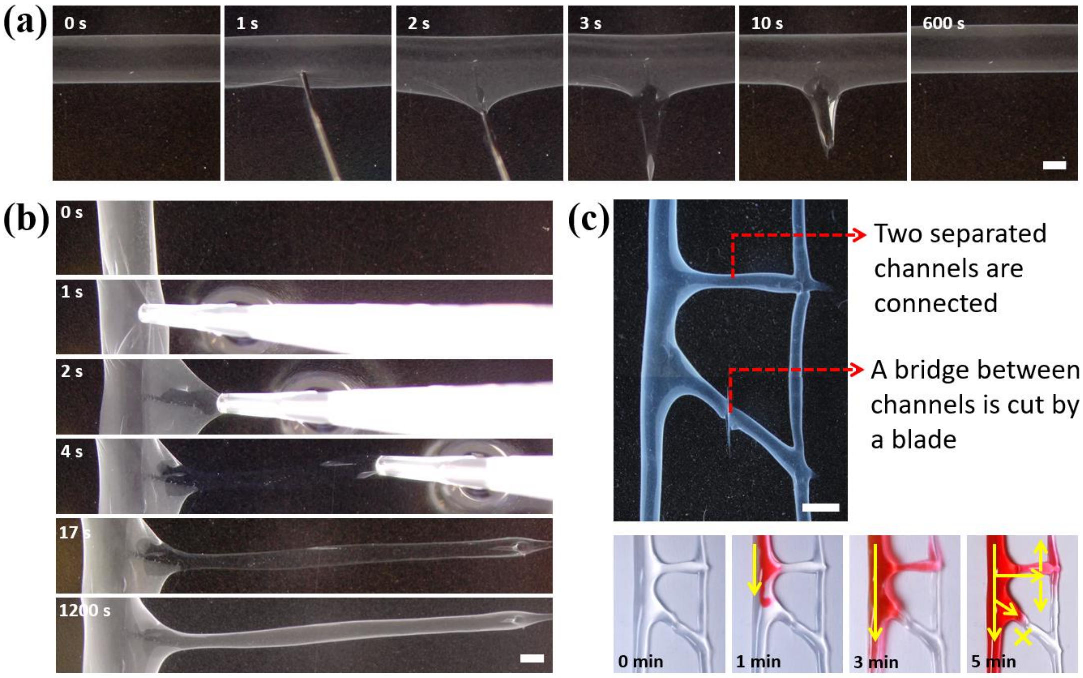

Supplementary Figure 8. Self-healing characteristics of membranous walls of printed chambers. (a) Membranes self-heal after being punctured by a sharp needle. Once the membranous complexes are punctured, chitosan ink inside the chamber can react with APAM matrix outside to generate new chitosan/APAM assemblies. The coacervation time is 30 minutes for the chamber in the image labeled 0 s. The scale bar is 1 mm. (b) New branches can be added to an established chamber, resulting from the self-healing properties of the membranous walls. The membrane assembly time is 30 minutes for the chamber in the image labeled 0 s. The scale bar is 1 mm. (c) Due to the self-healing properties of membranous walls, it is feasible to build new bridges between two separate chambers or to cut established bridges. Upon injecting dye liquids into the left-side channel, liquids can pass through the new bridge without leaking out, in comparison to the case when the channel has been truncated and liquid cannot flow through. The scale bar is 3 mm.

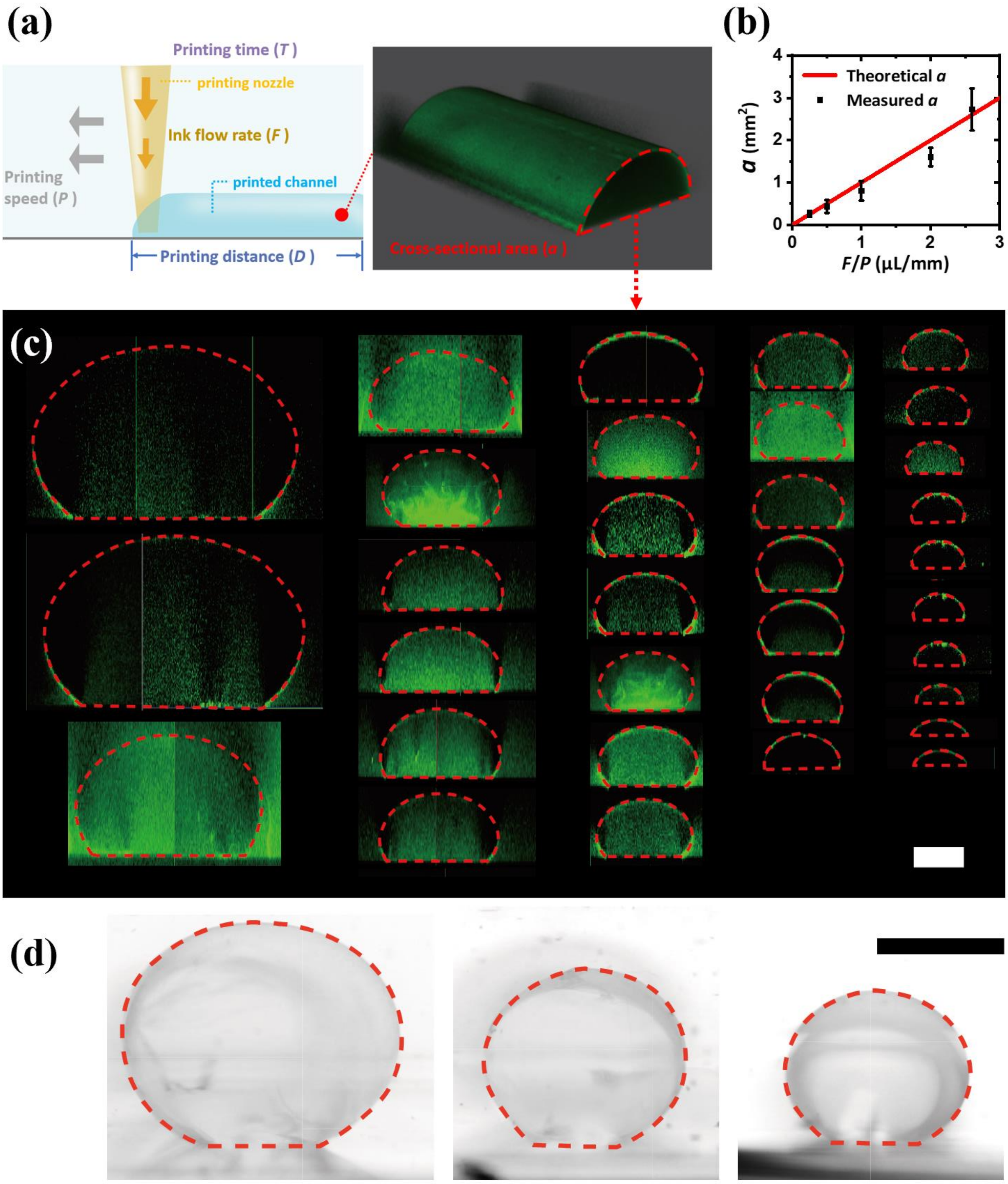

Supplementary Figure 9. (a) Illustration for printing of VasFluidic channels. (b) Relationship between printing ink flow rate F (μL s^{-1}), print speed P (mm s^{-1}), and the cross-sectional area a (mm^2) of the printed channel. Source data are provided as a Source Data file. (c) Confocal laser scanning microscope images showing cross sections of channels printed with different printing parameters. The printing ink is pre-mixed with 0.02 wt% FITC-Chitosan for visualization. Images are collected before removing the printing ink and matrix.

Boundaries of the cross-sections are highlighted with red dotted lines. The scale bar is 500 μm. (d) Photographs showing cross sections of channels with larger sizes. Boundaries of the cross-sections are highlighted with red dotted lines. The scale bar is 5 mm.

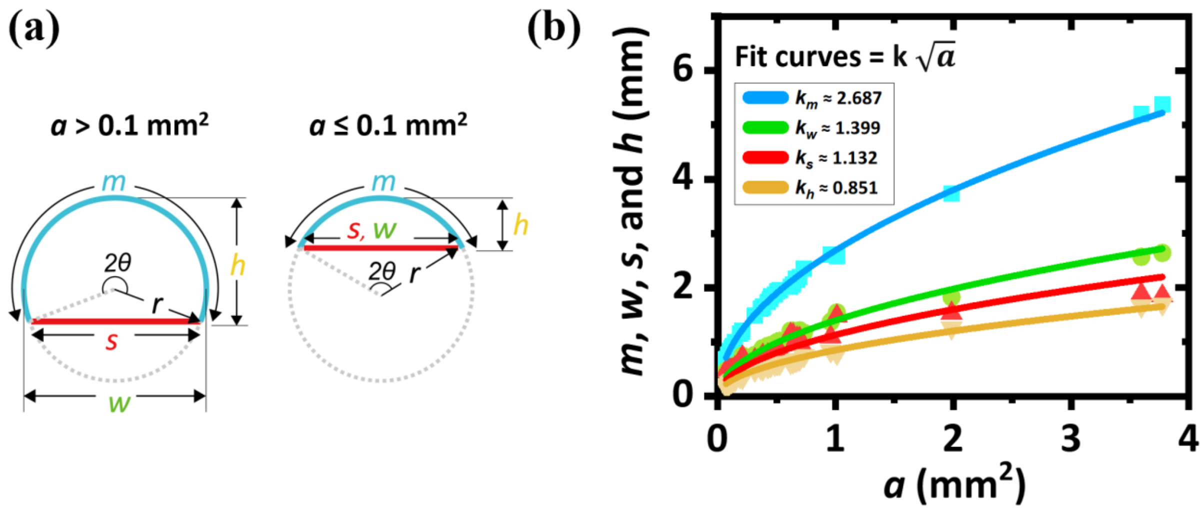

Supplementary Figure 10. Morphological parameters of channels' cross-sections. (a) Morphologies of cross-sections are approximated as parts of a circle with a radius of *r*. The cross-sectional area, the height, the maximum width, the length of the APAM/chitosan membrane part, and the length that channel attached to the substrate are defined as *a*, *h*, *w*, *m* and *s*, respectively. (b) Relation between *m* (or *w*, *s*, *h*) and *a*. The morphological parameters are measured with confocal laser scanning microscope images in Figure S9(c). The dots are measured data of *m* (blue), *w* (green), *s* (red), and *h* (orange), respectively. Source data are provided as a Source Data file. The solid lines are corresponding fit curves, and the functions of the fit curves are listed in Equation 4 in Supplementary Note S2. The measured data fit well with the fit curves, and thus *m* (or *w*, *s*, *h*) can be estimated with known values of *a*:

$m = 2.687\sqrt{a}$, $w = 1.399\sqrt{a}$, $s = 1.132\sqrt{a}$, $h = 0.851\sqrt{a}$.

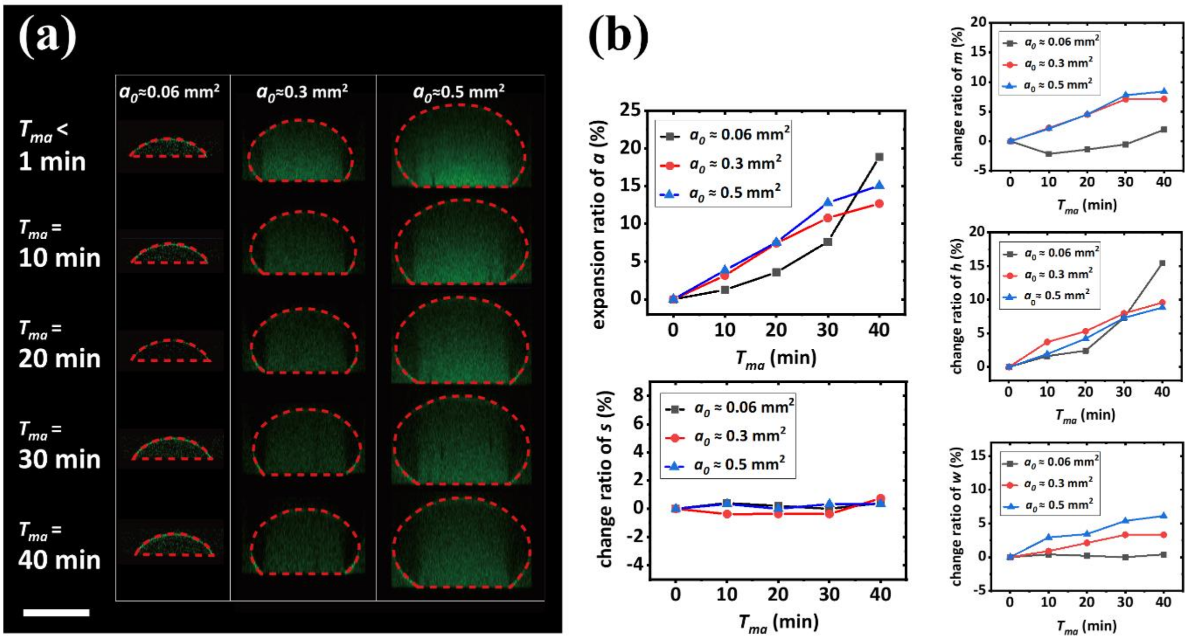

Supplementary Figure 11. The influence of self-assembly time (T_{ma}) on morphological parameters of cross-sections. The self-assembly starts when the printing process begins and is stopped by removing the APAM matrix. (a) Channels with different initial cross-sectional areas (a_0) are observed after printing. The scale bar is 500 µm. (b) The cross-sectional area (*a*), the height (*h*), the maximum width (*w*), and the length of the APAM/chitosan membrane part (*m*) has a change ratio of less than 10% when $T_{ma} \leq 20$ min. The length that channel attached to the substrate (*s*) has a change of less than 1% with the increasing T_{ma} . Source data are provided as a Source Data file.

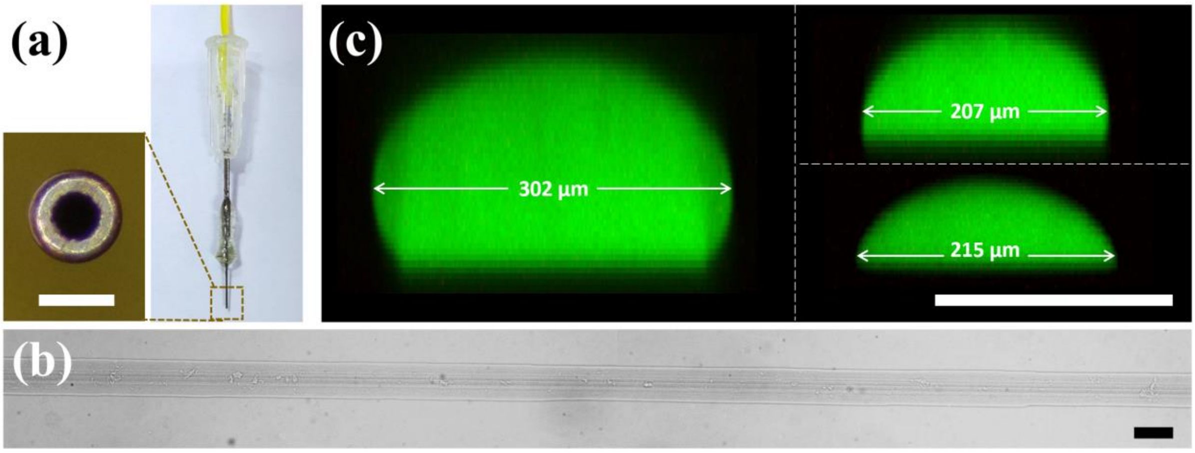

Supplementary Figure 12. Printing of thinner channels with smaller printing nozzle. We customized (a) a printing nozzle with a stainless steel needle as nozzle tip, and printed (b) a channel with width of around 200 µm. (c) Confocal laser scanning microscope images showing cross sections of 3 different sized channels printed with the stainless steel nozzle. All scale bars are 200 µm.

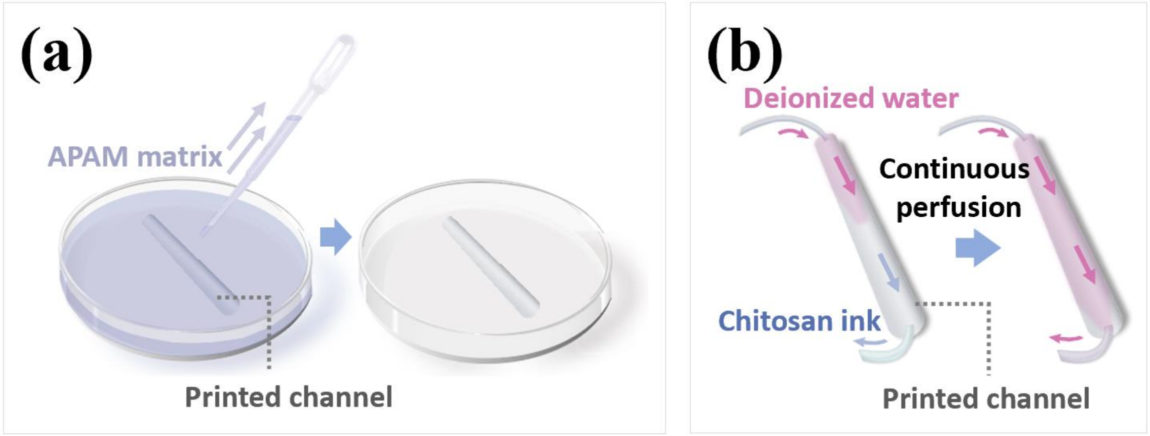

Supplementary Figure 13. Removal of APAM printing matrix and chitosan printing ink after the self-assembly of channel walls. (a) Use transfer pipettes or pipette tips to remove the APAM matrix gently. (b) Perfuse the channel with deionized water continuously to remove the chitosan ink inside.

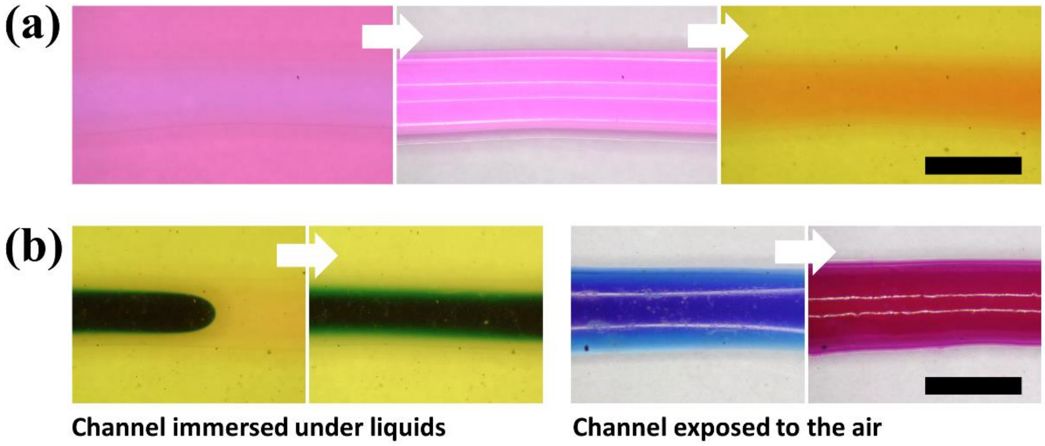

Supplementary Figure 14. (a) Refresh liquids outside the channel. (b) Refresh liquids inside the channel, which is immersed under liquids or exposed to the air. Scale bars are 2 mm.

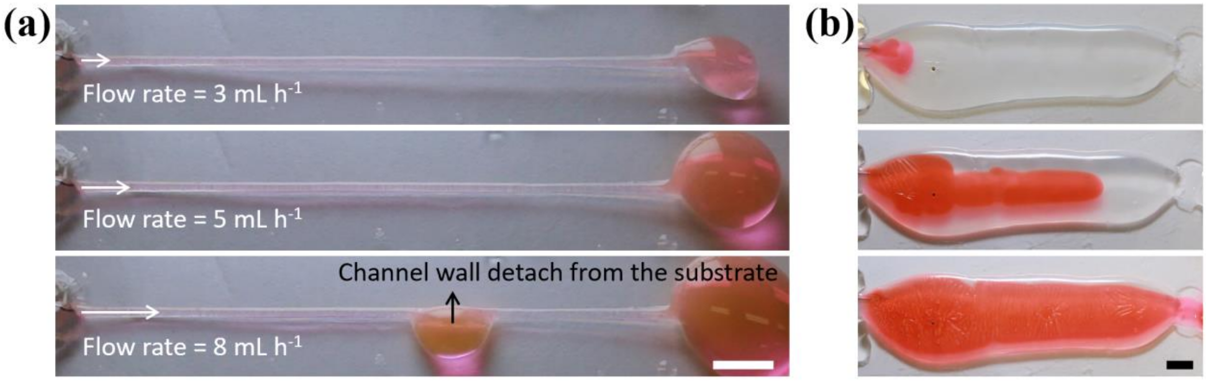

(c)

Channel Type	Channel width (mm)	Channel cross-sectional area (mm²)	Maximum flow rates (mL h⁻¹)	χ
Straight channel; 50-60 mm in length	0.6-0.7	0.21	6-8	0.12
	1	0.51	700-1000	1.57-2.25
	1.5	1.01	1380-1680	0.62-0.76
	2	1.98	> 1680 *	> 0.25

* Maximum flow rate for our pump is around 1680 mL h^{-1}, and the flow rate higher than 1680 mL h^{-1} is not applied here.

Supplementary Figure 15. Liquid perfusion of straight channels with different widths. 0.001-0.005 wt% rhodamine 6G aqueous solution is used as perfusion liquids for clear visualization. Channel outlets are exposed to air for smooth flow of internal liquids. The channel widths are measured before removing the printing matrix, corresponding to *w* shown in Supplementary Fig.10(a). (a) Liquid perfusion into a channel with width of 610 μm. The channel walls detach from the substrate as the flow rate reaches about 8 mL h^{-1}. The scale bar is 5 mm. (b) Liquid perfusion into a channel with width larger than 1 cm. The liquid flow rate is larger than 20 mL h^{-1}. The scale bars is 5 mm. (c) Maximum flow rates for 50-60 mm long straight channels with different widths, above which the channel walls will detach from the substrate. Channels are perfused under certain flow rates for at least 1 min to observe if liquid will leak out of the channel, or the channel walls will detach from the substrate. χ is a governing dimensionless number related to allowable flow rates in different sized channels, as explained in Supplementary Note 3.

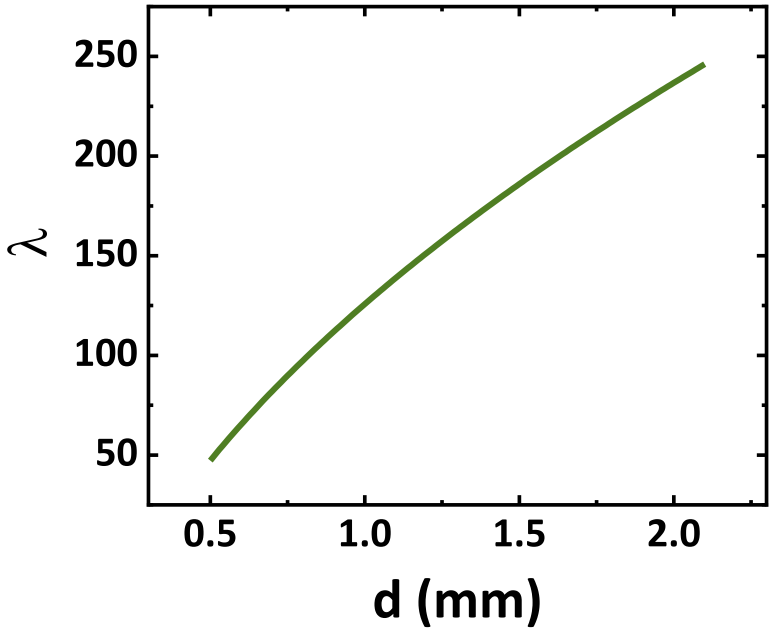

Supplementary Figure 16. Fitted curve based on the shape factor function $\lambda(d) = A\left(\frac{d}{d^*}\right)^{\gamma} + B$, which is defined in Supplementary Note 3. Experimental results in Supplementary Fig.15(c) are used for fitting. Source data are provided as a Source Data file.

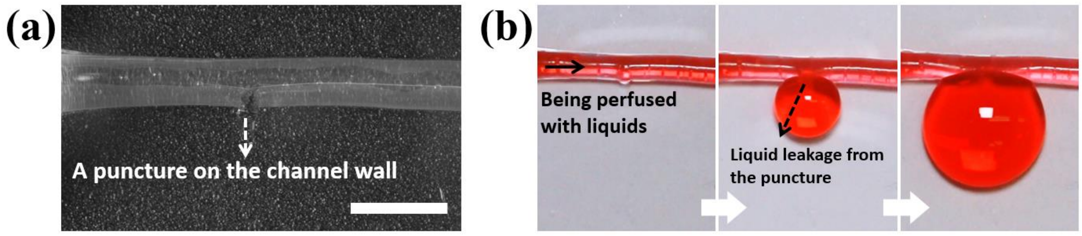

Supplementary Figure 17. The channel walls cannot self-heal after removing the printing ink and matrix. (a) A channel filled with water inside and exposed to the air. The channel wall is punctured with a sharp needle. The channel does not self-heal within 1 hour, which is confirmed by (b) perfusing the channel with dye solutions, during which the dye leaks out from the puncture. The scale bar is 5 mm.

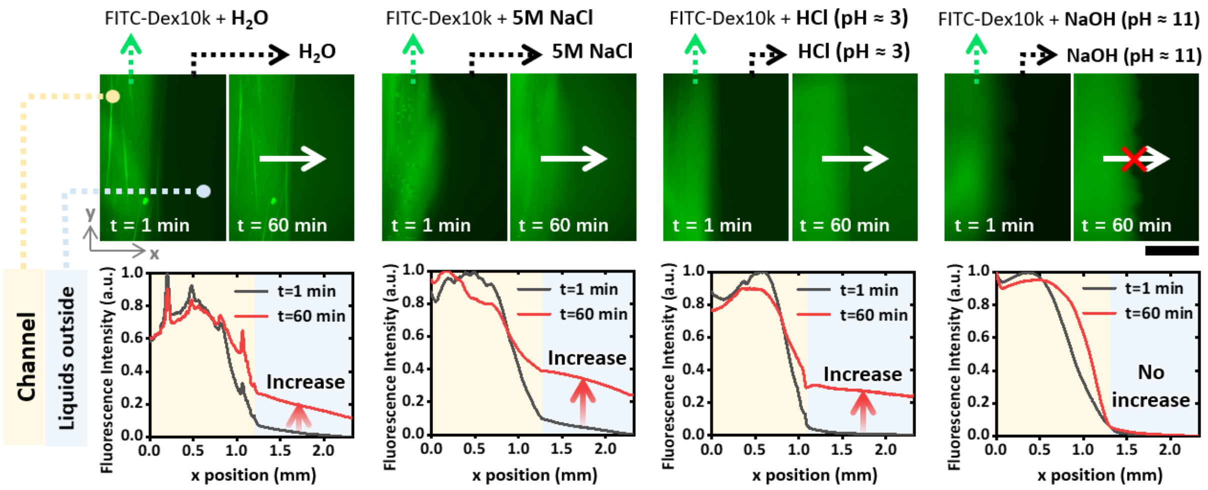

Supplementary Figure 18. Fluorescence microscope images showing the trans-wall transport of FITC-Dex10k ($R_h \approx 1.9$ nm) under hypersaline, acidic or alkaline environment. The permeability of channel walls increases under saline and acidic conditions, as more trans-wall FITC-Dex10k is observed within 60 min. The permeability of channel walls decreases with increasing pH, and FITC-Dex10k cannot cross the channel wall when increasing the pH to 11. The channel is infused with FITC-Dex solutions at a flow rate of 0.5 mL h^{-1}. Channel walls under view are exposed to 200 μL deionized water. Relative fluorescence intensities of different images in arbitrary units (a.u.) are analyzed with MATLAB. The scale bar is 1 mm. Source data are provided as a Source Data file.

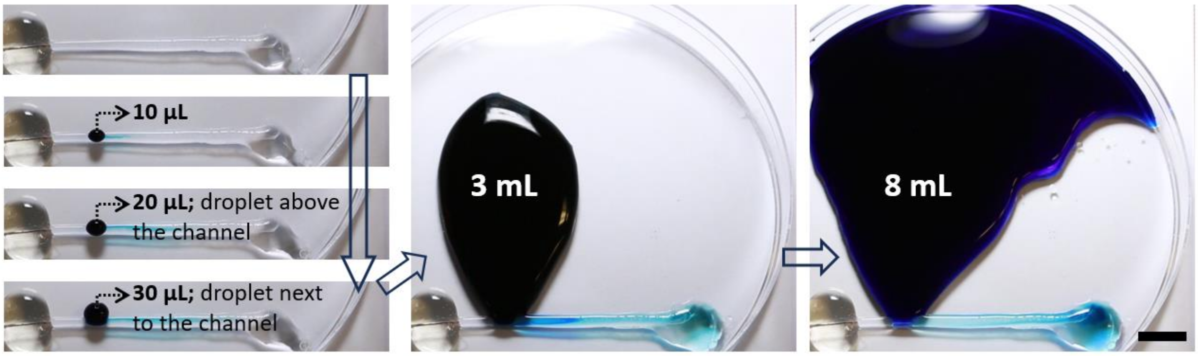

Supplementary Figure 19. Localized trans-wall introduction by depositing solutions on or next to the channel. The volume of the placed solution can be increased from microliters to milliliters. Methylene blue (MB) aqueous solution is used for easy visualization. Pipette tip is utilized to deposit the dye solution. The channel is infused with water inside at a flow rate of 2 mL h^{-1} during the deposition of MB solutions. The channel has been infused with water for at least 30 min before we place solutions next to the channel. The scale bar is 1 cm.

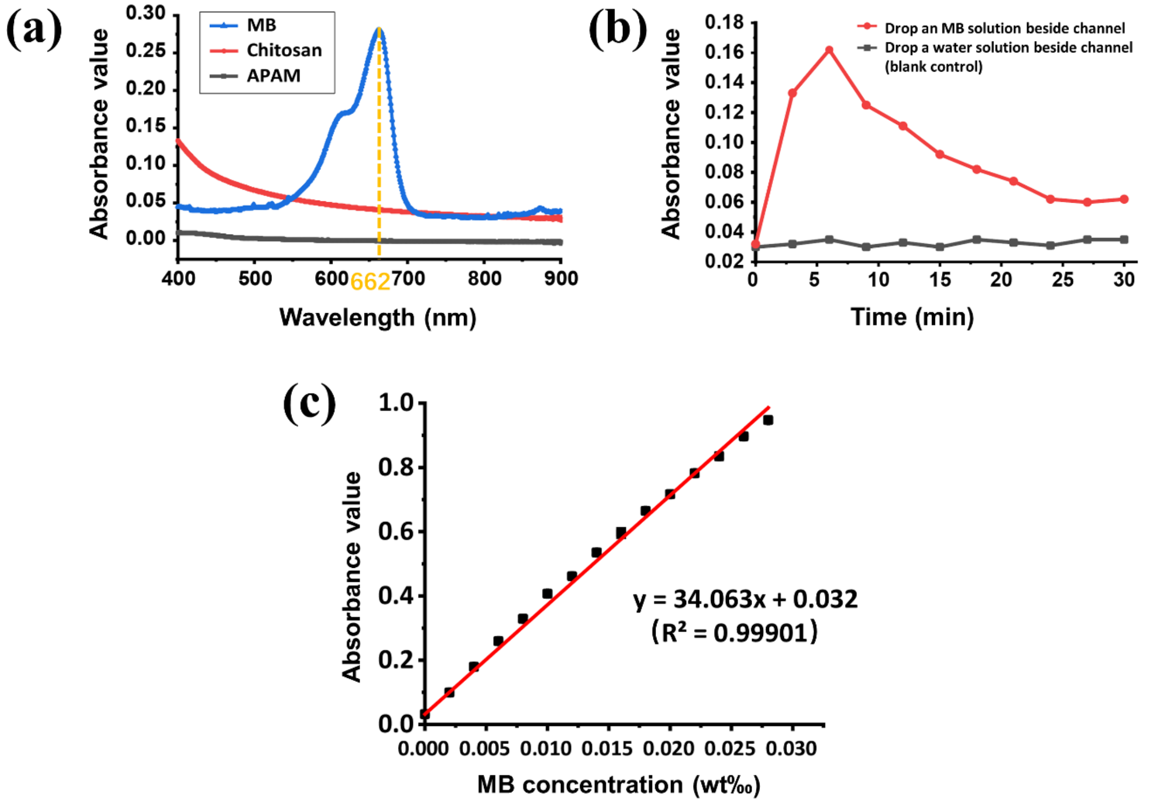

Supplementary Figure 20. Measurable concentration of trans-wall methylene blue (MB). (a) By analyzing the absorbance of solutions under a 662 nm wavelength, MB can be distinguished from the possible residual chitosan and APAM polymers after the removal of printing ink and matrix. The 662 nm wavelength is applied for the following detection of MB. (b) Absorbance of liquids inside after depositing an MB solution or a water solution on the channel. The trans-wall MB can vary the absorbance of liquids inside channel. The channel is perfused with deionized water inside (flow rate = 4 mL h^{-1}), and the downstream liquid is collected every 3 min for analyzing the absorbance. (c) MB concentrations in deionized water can be estimated by analyzing absorbance. Absorbance values and MB concentrations have approximate linearity when MB concentration is lower than 0.03wt‰. The fitting function (red line) between MB concentrations x and absorbance value y is $y = 34.063x + 0.032$. The corresponding coefficient of determination R^2 is 0.99901. Source data are provided as a Source Data file.

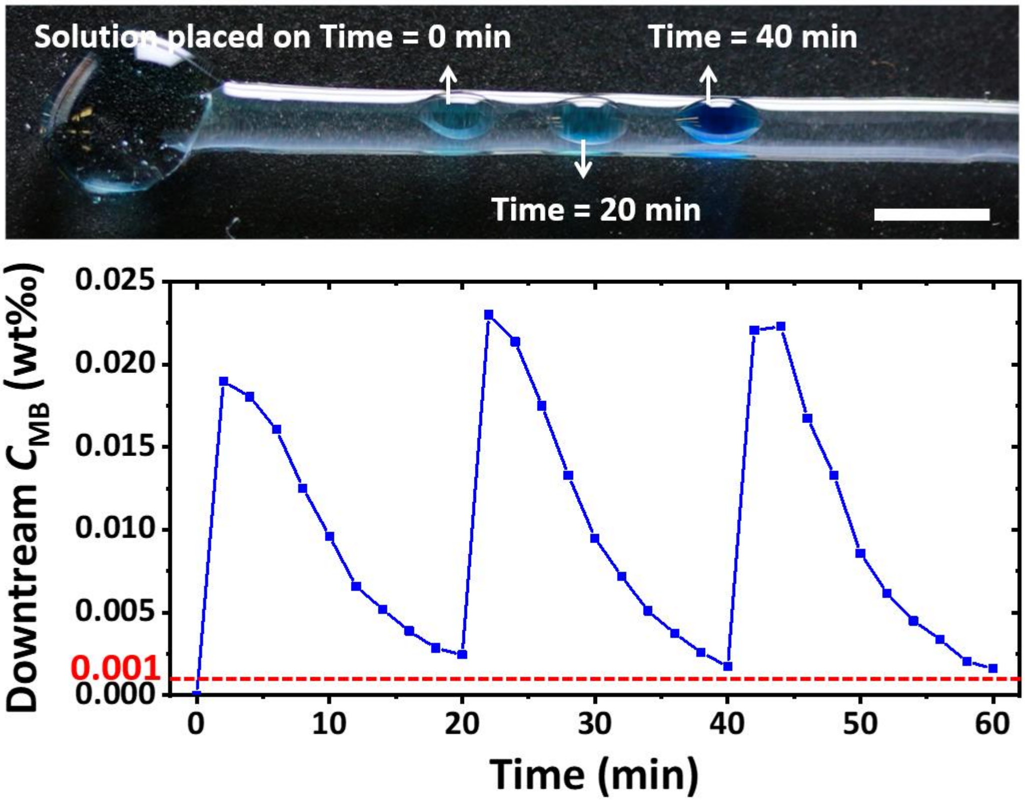

Supplementary Figure 21. MB concentration inside channel can be maintained above 0.001 wt ‰ by introducing 5 μL, 0.2 wt% MB solutions every 20 min. Similarly, the drug concentration in channel can also be maintained above a certain level by repeatedly introducing the drug via trans-wall transport. Source data are provided as a Source Data file.

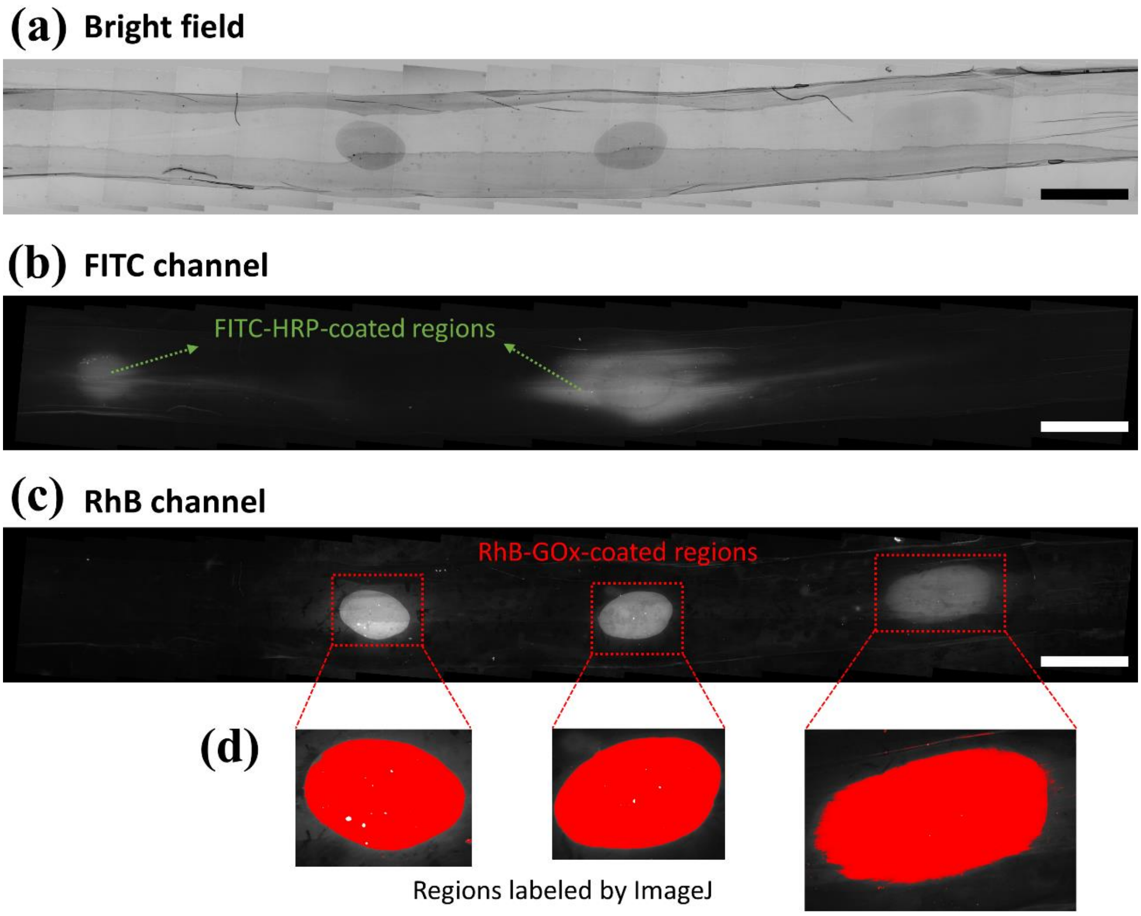

Supplementary Figure 22. An enzyme-coated channel under (a) bright field, (b) FITC channel, and (c) RhB channel. Fluorescence microscope images collected under the FITC channel and RhB channel show the regions coated with FITC-HRP and RhB-GOx, respectively. Scale bars are 2 mm. (d) Fluorescence microscope images under the RhB channel are used to analyze the relative fluorescence intensity of different RhB-GOx-coated regions. RhB-GOx-coated regions are labeled red by ImageJ for analysis.

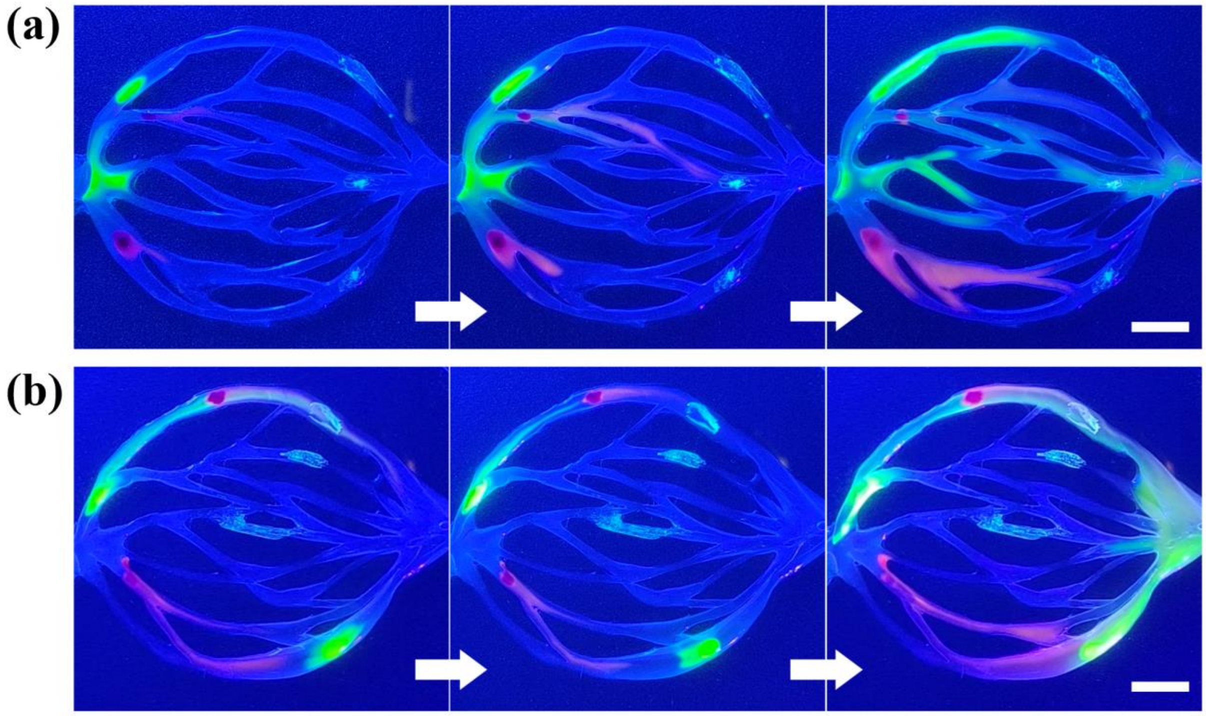

Supplementary Figure 23. Dye introduction and enzyme immobilization are localized at different regions of VasFluidics. (a) and (b) are optical images of 2 VasFluidic devices, which are perfused with deionized water inside and pre-modified with FITC-HRP and green fluorescent particles. Dye solutions are placed at different positions, passing through channel walls to change fluid compositions in different channel branches. Scale bars are 1 cm.

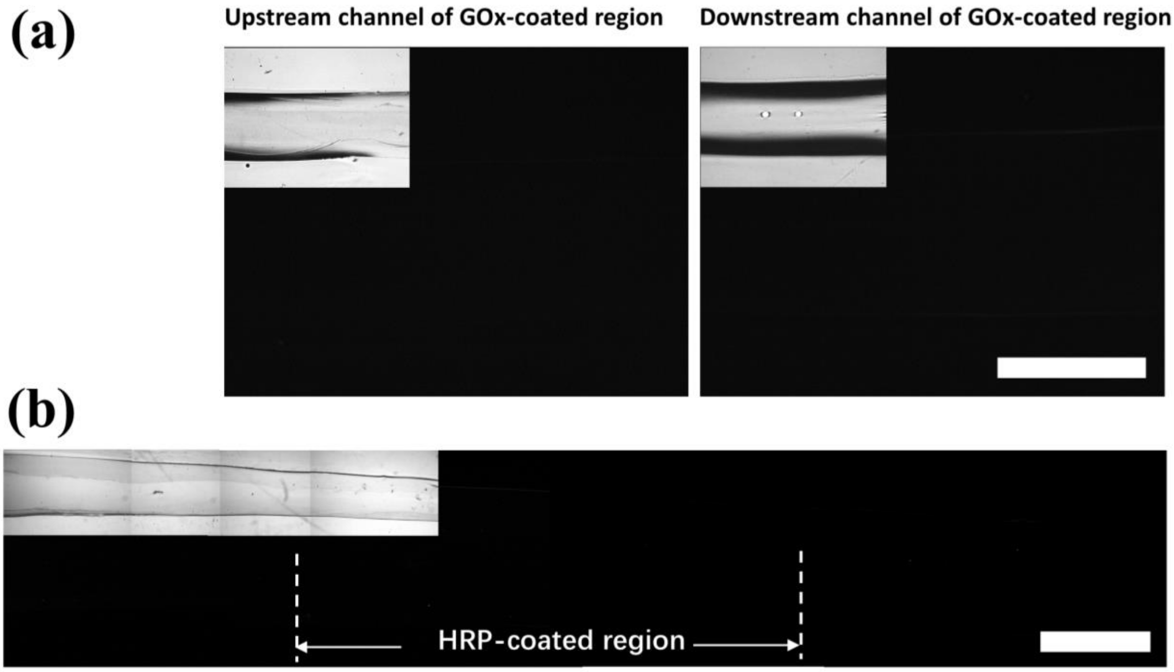

Supplementary Figure 24. Channels modified with GOx or HRP only as control groups; the bi-enzymatic cascade reaction for glucose degradation is not detected. (a) and (b) are optical and corresponding fluorescence microscope images of the channels. (a) A GOx-coated channel is perfused with aqueous solutions of glucose and Amplex Red. During the perfusion, no fluorescence is observed in the upstream or downstream channel of the GOx-coated region. (b) An HRP-coated channel is perfused with aqueous solutions of glucose and Amplex Red. During the perfusion, no fluorescence is observed in the channel. Scale bars are 1 mm.

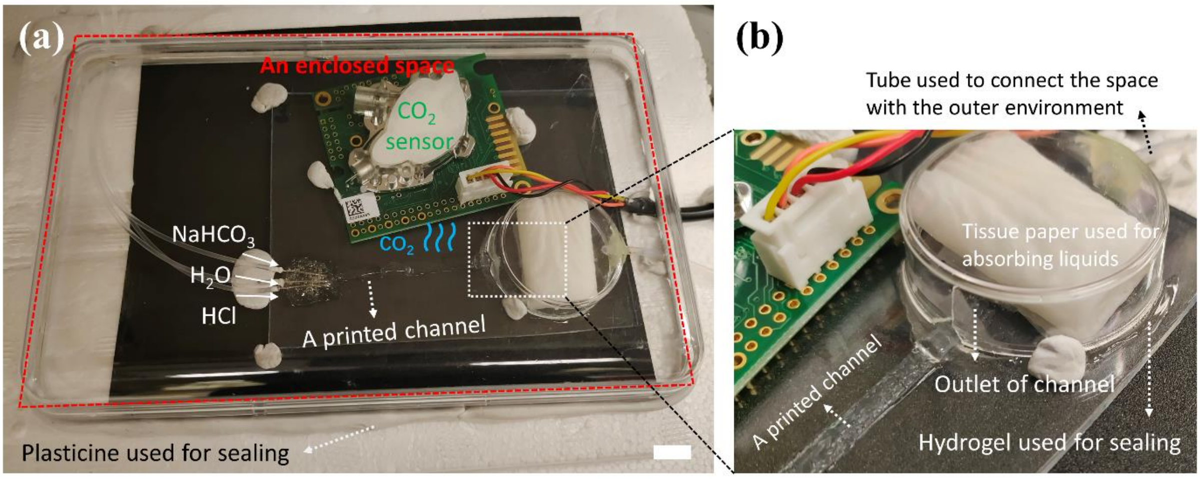

Supplementary Figure 25. Setups for detecting the carbon dioxide (CO_2) exhaled via trans-wall transport. (a) Channel in an enclosed space. The channel is perfused with deionized water, sodium bicarbonate solution ($NaHCO_3$, pH ≈ 9.7), and hydrochloric acid solution (HCl, pH ≈ 4). All liquids are perfused with a flow rate of 0.7 mL h^{-1}. A CO_2 sensor is placed beside the channel. The sensor and the channel are sealed in a transparent enclosed space with a volume of around 400 cm^3. The area of channel walls exposed to the air is around 4 cm × 4 mm. The outlet of the channel is sealed in (b) another enclosed space connected with the outer environment. Liquids flowing out from the channel outlet can be absorbed by tissue paper, and gas from the outlet can be expelled to the outer environment. The scale bar is 1 cm.

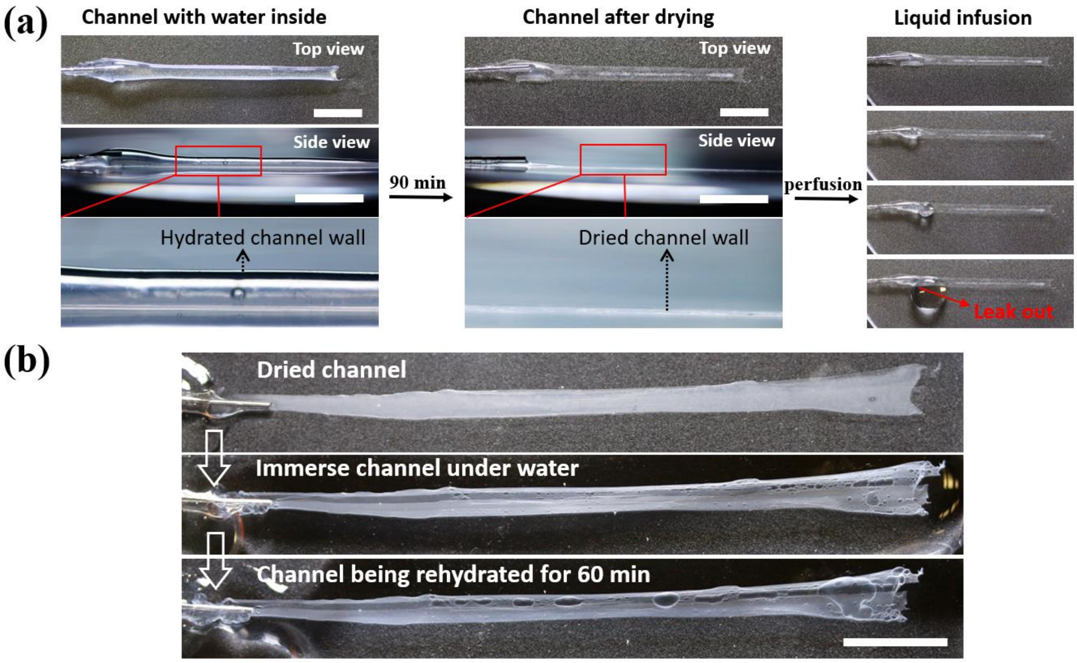

Supplementary Figure 26. (a) Liquids cannot be injected in the dried channel. (b) The dried channel cannot be restored to the original structure even after re-hydration. Scale bars are 1 cm.

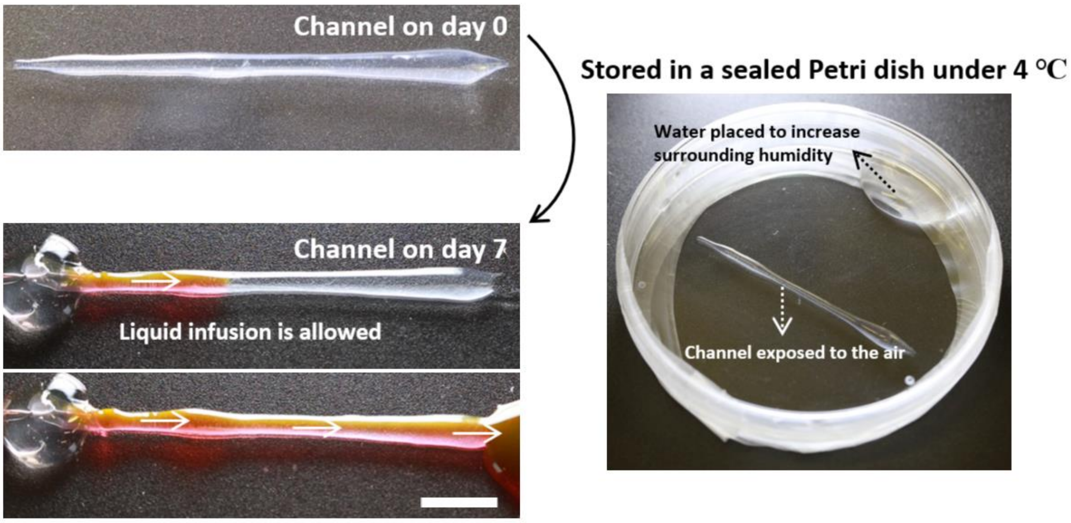

Supplementary Figure 27. Printed channel can be stored for 7 days, after which the channel still maintains the original structure and allows the infusion of liquids inside. During the storage in our experiments, APAM matrix outside the channel is removed and chitosan ink inside the channel is retained. Water is placed near the channel to increase the surrounding humidity, and thus the channel wall can stay hydrated. The channel is then stored in a sealed container and placed under 4°C. The scale bar is 1 cm.

Supplementary References

1. Grosskopf AK, Truby RL, Kim H, Perazzo A, Lewis JA, Stone HA. Viscoplastic matrix materials for embedded 3D printing. *ACS Appl. Mater. Interfaces* **10**, 23353-23361 (2018).

2. Lewis JA. Direct ink writing of 3D functional materials. *Adv. Funct. Mater.* **16**, 2193-2204 (2006).

3. Eggers J, Villermaux E. Physics of liquid jets. *Rep. Prog. Phys.* **71**, 036601 (2008).

4. Lewis JA, Gratson GM. Direct writing in three dimensions. *Mater. Today* **7**, 32-39 (2004).

5. Mustafa MB, Tipton DL, Barkley MD, Russo PS, Blum FD. Dye diffusion in isotropic and liquid-crystalline aqueous (hydroxypropyl) cellulose. *Macromolecules* **26**, 370-378 (1993).

6. Müller C, et al. Precise measurement of diffusion by multi-color dual-focus fluorescence correlation spectroscopy. *Europhys. Lett.* **83**, 46001 (2008).

7. Yuan W, Lv Y, Zeng M, Fu BM. Non-invasive measurement of solute permeability in cerebral microvessels of the rat. *Microvasc. Res.* **77**, 166-173 (2009).

8. Courjean O, Gao F, Mano N. Deglycosylation of glucose oxidase for direct and efficient glucose electrooxidation on a glassy carbon electrode. *Angew. Chem.* **121**, 6011-6013 (2009).

9. Tan S, Gu D, Liu H, Liu Q. Detection of a single enzyme molecule based on a solid-state nanopore sensor. *Nanotechnology* **27**, 155502 (2016).

10. Schultz SG, Solomon A. Determination of the effective hydrodynamic radii of small molecules by viscometry. *J. Gen. Physiol.* **44**, 1189-1199 (1961).

11. Pérez S, Bertoft E. The molecular structures of starch components and their contribution to the architecture of starch granules: A comprehensive review. *Starch-Stärke* **62**, 389-420 (2010).
