# Supplementary material for: Vascular network-inspired fluidic system (VasFluidics) with spatially functionalizable membranous walls
Source: Nat Commun. 2024 Feb 16;15:1437. doi: 10.1038/s41467-024-45781-3 (PMC10873510; doi:10.1038/s41467-024-45781-3)
Supplement: Supplementary file 3 — Description of Additional Supplementary Files [file 41467_2024_45781_MOESM3_ESM.pdf]

Supplementary Movies

Supplementary Movie 1: Channel with soft and elastic walls.
The channel can deform under external force and resume the original shape upon removal of the force. The external force is applied by using a pipette tip. The scale bar is 1 mm.

Supplementary Movie 2: Liquid infusion into printed channel
Infusion of rhodamine 6G aqueous solution into a vascular network-shaped channel. The scale bar is 5 mm.

Supplementary Movie 3: Channel with variable intracavity volume
The fluidic channel with soft walls can be inflated or collapsed to alter intracavity volume in response to the changes in liquid volume inside. The channel collapses when liquids inside flow out, and inflates as the liquid volume inside increases. The scale bar is 2 mm.

Supplementary Movie 4: Dye extraction from channel and dye introduction into channel
Liquid components within a Y-shaped channel are spatiotemporally regulated by localizing the trans-wall transport of specific dye molecules. When a water drop is deposited on the channel, local dyes inside are extracted and diffused into the droplet. The introduction of methylene blue (MB, a blue dye) into the channel is also localized by attaching an MB drop to the channel. The scale bar is 1 cm.

Supplementary Movie 5: Channel with localized functional regions
A VasFluidic channel where 4 downstream regions are immobilized with enzymes, and 4 upstream regions are designated for dye introduction. The scale bar is 1 cm.
